# German Literature

**Published:** 1870-08

**Authors:** 


### GERMAN LITERATURE.

The young ladies of New York city are acquiring distinction as German scholars. Miss Nannie Brooks, daughter of Hon. Erastus Brooks, Editor of the *New York Evening Express*, speaks the German language more correctly and writes and composes with greater accuracy than any other native American, while a correspondent of the *New York Independent* says, in a late letter, " among the seven hundred American residents in Dresden, Miss Mary, daughter of the Editor of the *Journal of Health*, is one of the very best German scholars, and on her return home will be well qualified to take a high position in German literature.

The study of the German language, being the language of philosophy and philology, is becoming very common among educated young ladies in New York. It is the language of thought, as French is of compliment. Germany swarms with Americans and English. Travellers, especially ladies, will find a delightful home at Heidelberg, at the house of Frau J. G. Foester, No. 58 Anlage, the Fifth Avenue of the place. The charges are about a paper dollar a day, and those who bear letters of introduction to the Frau, will be delighted with all the surroundings.

Gentlemen will find, at the hotel of Herr Lang, six Academie Strasse, good attendance, moderate bills, and a gentlemanly host, who speaks fluently German, English, and French. Fortunate will be the weary traveller who can procure for himself and family a resting-place at the house of Mrs. Hughes, 34 Prager Strasse, Dresden, where will be found an American table, most bountifully spread, with a lady at its head, of refinement and culture, and all surroundings of a like indication.

